# Effect of Second Language Proficiency on Inhibitory Control in the Simon Task: An fMRI Study

**DOI:** 10.3389/fpsyg.2022.812322

**Published:** 2022-02-21

**Authors:** Fanlu Jia

**Affiliations:** School of Education and Psychology, University of Jinan, Jinan, China

**Keywords:** bilinguals, proficiency, inhibitory control, Simon task, fMRI

## Abstract

How learning a second language (L2) changes our brain has been an important question in neuroscience. Previous neuroimaging studies with different ages and language pairs spoken by bilinguals have consistently shown plastic changes in brain systems supporting executive control. One hypothesis posits that L2 experience-induced neural changes supporting cognitive control, which is responsible for the selection of a target language and minimization of interference from a non-target language. However, it remains poorly understood as to whether such cognitive advantage is reflected as stronger controlled processing or increased automatic inhibition processing. In this study, using functional MRI we scanned 27 Chinese-English late bilinguals while they performed a Simon task. Results showed that bilinguals with higher L2 vocabulary proficiency performed better in the Simon task, and more importantly, higher L2 vocabulary proficiency was associated with weaker activation of brain regions that support more general cognitive control, including the right anterior cingulate cortex, left insula and left superior temporal gyrus. These results suggest that L2 experience may lead to a more automatic and efficient processing in the inhibitory control task. Our finding provides an insight into neural activity changes associated with inhibitory control as a function of L2 proficiency.

## Introduction

Human being has a remarkable ability to learn more than one language, with which even an older adult can learn a second language with success. A wide range of bilingual studies showed that both first and second languages were activated when a bilingual uses one of them (Smith, 1997; Brysbaert, 1998; Francis, 1999; Bialystok et al., 2004; Conrad et al., 2015). The joint activation of both languages suggests that bilinguals must control attention to the selected languages in order to achieve fluent performance in the designated language without interference from the other language. This situation is similar to that encountered in the inhibitory control problems, in which cognitive resources are focused on goal-relevant processing while filtering out irrelevant information that can interferes with the appropriate response (Shallice et al., 1996; Green, 1998; Van Heuven et al., 1998; Garbin et al., 2010).

Considerable evidence has linked regular use of two languages with better ability of cognitive control (Bialystok et al., 2005). Previous studies have shown that bilinguals were more skilled than monolinguals in performing tasks that require attentional control to ignore or inhibit interference information (Bialystok, 2001; Bialystok et al., 2004). For example, using the Simon task, in which conflict arises from the mismatch between stimuli location and the direction of response, Bialystok et al. (2004) found that bilinguals across lifespan performed the task more quickly than monolinguals and showed less interference from the position information in the incongruent condition (Bialystok et al., 2004). They proposed that bilingual advantage emerged in bilinguals’ control processes, which enables switches between two-language systems, and are responsible for the selection of a target language and minimization of interference from a non target language (Costa et al., 2006; Mohades et al., 2014).

By now, mixed results have been obtained with regard to the effect of bilingualism on the brain cognitive functions. Greater activation for bilinguals contrasted with monolinguals has been found in the brain regions critical for conflict processing (Bialystok et al., 2005; Mohades et al., 2014), though some studies have reported a reversed pattern (Abutalebi et al., 2012). In a magneto-encephalography study (Bialystok et al., 2005), brain activation of 30 adults –Cantonese-English bilinguals, French-English bilinguals, and English monolingual – were recorded while performing the Simon task. The two bilingual groups showed faster response and greater activity in superior and middle temporal, cingulate, and superior and inferior frontal regions. In an fMRI study by Mohades et al. (2014), brain activation in performing the Simon task was compared across three groups of 8–11-year-old-children, i.e., bilinguals from birth (2 L1), second language learners (L2L), and monolinguals (1 L1). They found significantly higher activities of caudate nucleus, posterior cingulate gyrus, superior temporal gyrus (STG) and precuneus in incongruent condition relative to congruent condition in bilingual children compared to monolingual peers. Moreover, greater activation was found in the brain regions underlying nonverbal conflict processing, verbal conflict processing, and language processing in 2 L1 compared to L2L (Mohades et al., 2014). In contrast, in anther fMRI study, Abutalebi et al. (2012) used language-switching and flanker tasks, and found less activity for bilinguals than monolinguals in the anterior cingulate cortex (ACC) during conflict processing. They suggested that the bilingual brain may adapts better to resolve cognitive conflict and thus bilinguals require less neural resources to perform domain-general cognitive tasks.

Previous behavioural and neuroimaging studies have examined the impact of L2 learning on cognitive control abilities predominantly by comparing behavioural performance or brain activation between bilinguals and monolinguals. However, it remains poorly understood as to the relationship of L2 proficiency and conflict processing in the bilingual brain. One possibility is that higher proficient bilinguals use more neural resources to deal with conflicts situation such that greater activation for conflict processing would be observed. Alternatively, constantly encountering and resolving language conflicts in higher proficient bilinguals may lead to more automatic inhibition processing in conflict situation, considering that automatic responding can develop with enough training (Schneider and Shiffrin, 1977; Verbruggen and Logan, 2008). This leads to a prediction of a negative correlation between L2 proficiency and brain activation in the inhibitory control tasks. To address this question, we scanned 27 late Chinese-English bilinguals while they performed a Simon task using fMRI. We performed correlation analyses between the subjects’ L2 proficiency and their brain activation in the task.

## Materials and Methods

### Subjects

We scanned 28 adults (17 males and 11 females, average age 41 y and 10 mo, ranging from 30 y 1 mo to 52 y 11 mo), who were native Chinese speakers and learned English as a second language. The subjects completed a language-background questionnaire (Li et al., 2014). The language usage chart addressed the usage frequency of each language at home, at work, with friends, and overall. The responses indicate the extent to which each language is used daily and the degree to which the participant is functionally bilingual. And they spent much more time using Chinese (L1) than L2 in their daily life. They started to learn L2 later than 8-year-old (mean age 11.9 y with standard deviation at 1.8 y). The subjects were physically healthy and free of neurological disease, head injury, and psychiatric disorder. All the participants had completed college education and experienced 15–21 years school education (mean 18.1 y with standard deviation at 2.2 y). They also reported that they acquired 2–15 years English education (mean 8.4 y with standard deviation at 3.4y). The participants came from different fields (i.e., teachers, company employees, doctors and civil servants and lived in the same city). The study was approved by the ethical committee of the Beijing MRI Center for Brain Research, Chinese Academy of Sciences, and informed consent was obtained from all subjects. All of the subjects were right-handed as assessed by a handedness inventory, with handedness scores higher than 33 (Snyder and Harris, 1993). Subjects had normal or correct-to-normal vision.

### Design and Materials

An English word reading test was used to measure subjects’ current level of L2 (see also Tan et al., 2011). Previous studies have measured its reliability as a predictor of L2 proficiency in adults (Laufer, 1998; Schmitt et al., 2001; Zareva et al., 2005). The vocabulary is usually regard as “the building block of language” (Schmitt et al., 2001), and it is considered by some to be “the single most important aspect of foreign language learning” (Knight, 1994). Meanwhile, a model has been proposed, which could virtually explain all the variance in the vocabulary knowledge of learners at different levels of language proficiency (Zareva, 2005). Several studies have shown that tests of breadth and depth of L2 vocabulary knowledge could very well predict success in reading, writing, general proficiency and academic achievement (Saville-Troike, 1984; Grabe, 1991; Qian, 1999; Laufer and Goldstein, 2004). The test was composed of 135 English words, among which 115 were selected according to Public English Test System (PETS) in China and the other 20 low-frequency items were from a language corpus (i.e., Graduate Record Examination). The numbers of words from PETS-1 to PETS-4/5 textbooks were 30, 30, 30, and 25, respectively. Words were arranged in a list from easy to difficult based on PETS level. Participants were asked to read the words aloud as quickly and accurately as possible if he/she knew them. The test would stop until three consecutive errors or no-reply. Their reading scores are illustrated in Table 1.

**Table 1:** Demographic characteristics and behavioral results.

Variable	Results
Age, yr	41.4 (8.2)
No. of subjects	
No. of males	17
No. of females	10
Decision accuracy, %	
Decision accuracy in incongruent condition, %	81.7% (17.3%)
Decision accuracy in congruent condition, %	88.0% (10.3%)
Decision accuracy in neutral condition, %	87.8% (12.8%)
Decision RT, ms	
Decision RT in incongruent condition, ms	391 (67.5)
Decision RT in congruent condition, ms	378 (34.1)
Decision RT in neutral condition, ms	395 (45.3)
English word reading scores	
L2 reading (max = 135)	81.1 (43.1)

SDs are given in parentheses.

The subjects performed a Simon task in the scanner, in which red or green squares were visually presented on the left or right side of the screen. Participants were instructed to press left response key if a red square appeared and right response key if a green square was shown, irrespective of its position. Participants were instructed to respond as quickly as possible. The response keys were placed comfortably one under each hand and participants placed each index finger over one of the keys. Congruent trials were those in which the correct response key was on the same side as the stimulus and incongruent trials were those in which the reverse was true. In a neutral condition, the same stimuli were presented in the center of the screen. The response rule that connected the stimulus color to the response key was the same, but because the stimuli were always presented centrally, there was no conflicting position information.

An event-related design was used. The experiment was conducted within a single run. Each trial began with a fixation cross shown in the center of the screen for 200 ms. The stimulus then appeared for 600 ms on the left or right side or in the center, followed by a 1,200-ms blank interval. Each trial lasted for 2000 ms. There were 48 trials in each of the three conditions, resulting in 144 trials in total. The experiment ended with a 2 s fixation period. Prior to the scanning, all subjects had some practice to familiarize with task procedures.

#### MRI Acquisition

Whole-brain imaging data were acquired using a 3 T Siemens MRI scanner at the Beijing MRI Center for Brain Research of the Chinese Academy of Sciences. T2^*^-weighted gradient-echo echo planar imaging (EPI) sequence was used [echo time (TE) = 30 ms, repetition time (TR) = 2 s, flip angle = 90°, field of view = 22 cm, slice thickness = 4 mm, and the image matrix = 64 × 64]. Thirty-two contiguous axial slices were acquired to cover the whole brain. Visual stimuli were presented to subjects through a projector onto a translucent screen. Subjects viewed the stimuli through a mirror attached to the head coil. High-resolution (1 × 1 × 1 mm^3^) anatomical images were acquired using a T1-weighted, 3-D gradient-echo sequence.

### Data Analysis

#### Behavioral Data Analysis

We examined the accuracies of all subjects and discovered that most of subjects had higher accuracy rate except one. This subject was excluded from the sample since she did not make any judgment on the first 42 trials. There were 27 subjects in the final sample. The accuracy of the responses and the response times (RT) were compared across conditions and were correlated with English reading test score and the age of acquisition for English, a variable which has been correlated with the executive function. These analyses were performed using the Statistical Package for Social Sciences 22.0.

#### Image Data Analysis

SPM8 were used for image preprocessing and statistical analyses. The functional images were realigned and resliced to remove movement-by-susceptibility induced variance. They were then spatially normalized to an EPI template based on the ICBM152 stereotactic space, an approximation of canonical space and spatially smoothed using an isotropie Gaussian kernel (8-mm full width at half-maximum). Individual subject’s activation t map was generated by using the general linear model in which time series were convolved with the canonical hemodynamic response function and were high-pass-filtered at 128 s. Individual incongruent conditions versus neutral conditions contrast images were used in a random effects model to create a group-level statistical map, with the voxel wise threshold set at *p* < 0.05, FDR corrected for multiple comparisons, and an extent threshold of 10 congruous voxels. The right middle frontal gyrus (MFG, BA6, *x* = 42, *y* = −6, *z* = 52), right ACC (BA24/32, *x* = 12, *y* = 4, *z* = 50), left and right insula (BA13, *x* = −44, *y* = −14, *z* = 22; *x* = 46, *y* = 12, *z* = 0), left STG (BA22, *x* = −48, *y* = −16, *z* = −2) and left posterior parietal cortex (PPC, BA7, *x* = −22, *y* = −38, *z* = 56) were defined as regions of interest (ROIs, 6 mm box) in the all-participant incongruent > neutral activation map that survived the threshold of *p* < 0.05 FDR corrected. Previous studies showed that these regions were associated with inhibitory processing (MacDonald et al., 2000; Maclin et al., 2001; Peterson et al., 2002; Bialystok et al., 2005). To identify brain regions showing significant correlation between cortical activation underlying conflict processing and L2 proficiency, the average blood-oxygen-level-dependent contrast estimates of the voxels within the six ROIs were extracted for each subject, and Pearson’s r correlations were performed between ROIs’ activation levels and subjects’ L2 reading scores.

## Results

### Behavioral Results

Average response accuracies and reaction times (RTs) across subjects were 81.7%/391 ms, 88.0%/378 ms and 87.8%/395 ms for incongruent, congruent and neutral conditions, respectively (Table 1).

**Figure 1:**
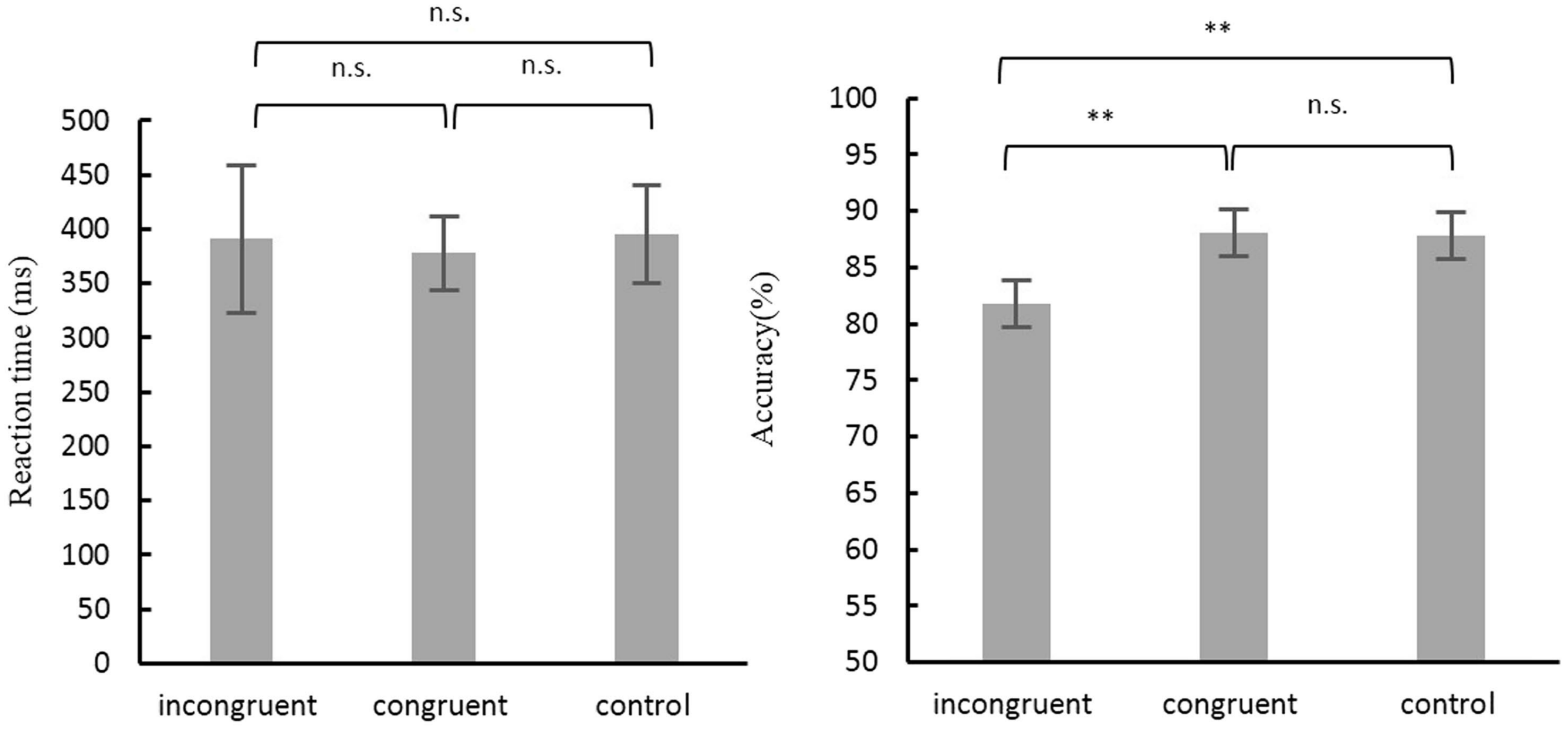
Accuracy and Reaction time for different conditions in Simon task. Error bars depict SD. n.s., not significant; ^**^p < 0.01.

The mean accuracy rate of the Simon task was analyzed with a repeated measures ANOVA with Condition (3 levels) as a within-subjects factor. The results revealed a significant main effect of Condition [*F*(2, 52) = 9.20, *p* < 0.001]. The Bonferroni-corrected *post hoc t*-tests revealed a significant difference between incongruent and congruent conditions (*p* < 0.01), and also between incongruent and neutral conditions (*p* < 0.01). As for the mean RT, results showed no significant effect for the Condition type, *F*(2, 52) = 2.205, *p* = 0.120 (Figure 1).

**Figure 2:**
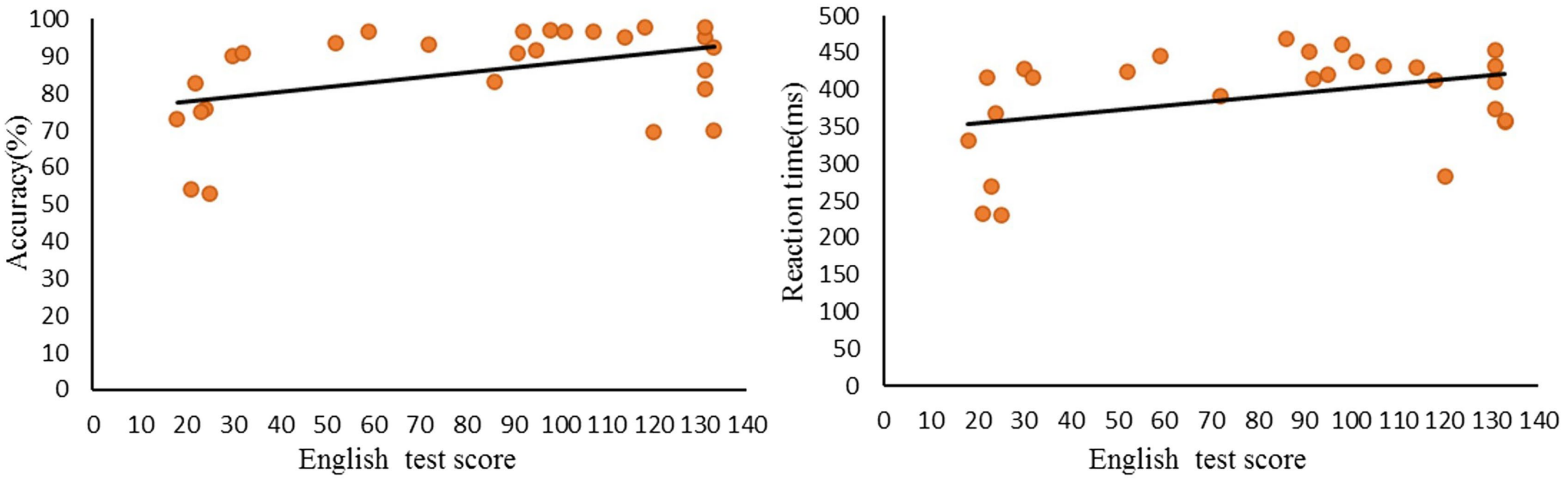
Correlations between L2 reading scores and Accuracy/Reaction time during incongruent judgment.

Moreover, we found a significant positive correlation (*r* = 0.439, *p* < 0.05) between English test scores and accuracy rates in the incongruent condition. However, RTs showed no significant correlation (*r* = 0.375, *p* = 0.054) with English test scores (Figure 2).

Despite of that, we found negative correlation (*r* = −0.475, *p* < 0.05) between ages of acquisition English and accuracy rates in the incongruent condition. The RTs in the incongruent condition was not correlated with age of acquisition for English (*r* = −0.321, *p* = 0.102). Partial correlation analyses were conducted between ages of acquisition English, accuracy rates and RTs in the incongruent condition, controlling for the effects of age. The correlation related to accuracy rates remained statistically significant when ages was controlled. The partial correlation coefficient was −0.475 (*p* < 0.05) for accuracy rates and − 0.317 (*p* = 0.114) for RTs. And we did not discover correlation between the predictors of conflict processing (i.e., accuracy rates and RTs in the incongruent condition), ages of acquisition English and ages. The correlation coefficient was −0.027 (*p* = 0.892) for accuracy rates, −0.074 (*p* = 0.715) for RTs and 0.081 (*p* = 0.687) forages of acquisition English. The results provided evidence for the impact of age of L2 acquisition on conflict processing.

In addition, we examine the effect of education on the inhibitory processing. The education period (from primary school to college) showed no correlations with RTs (*r* = 0.287, *p* = 0.146) and accuracy rates (*r* = 0.339, *p* = 0.084) in the incongruent condition. The English education period showed no correlations with RTs (*r* = 0.343, *p* = 0.080). The correlation coefficient between English education period and accuracy rates failed to reach significance (*r* = 0.378, *p* = 0.052).

### fMRI Results

**Figure 3:**
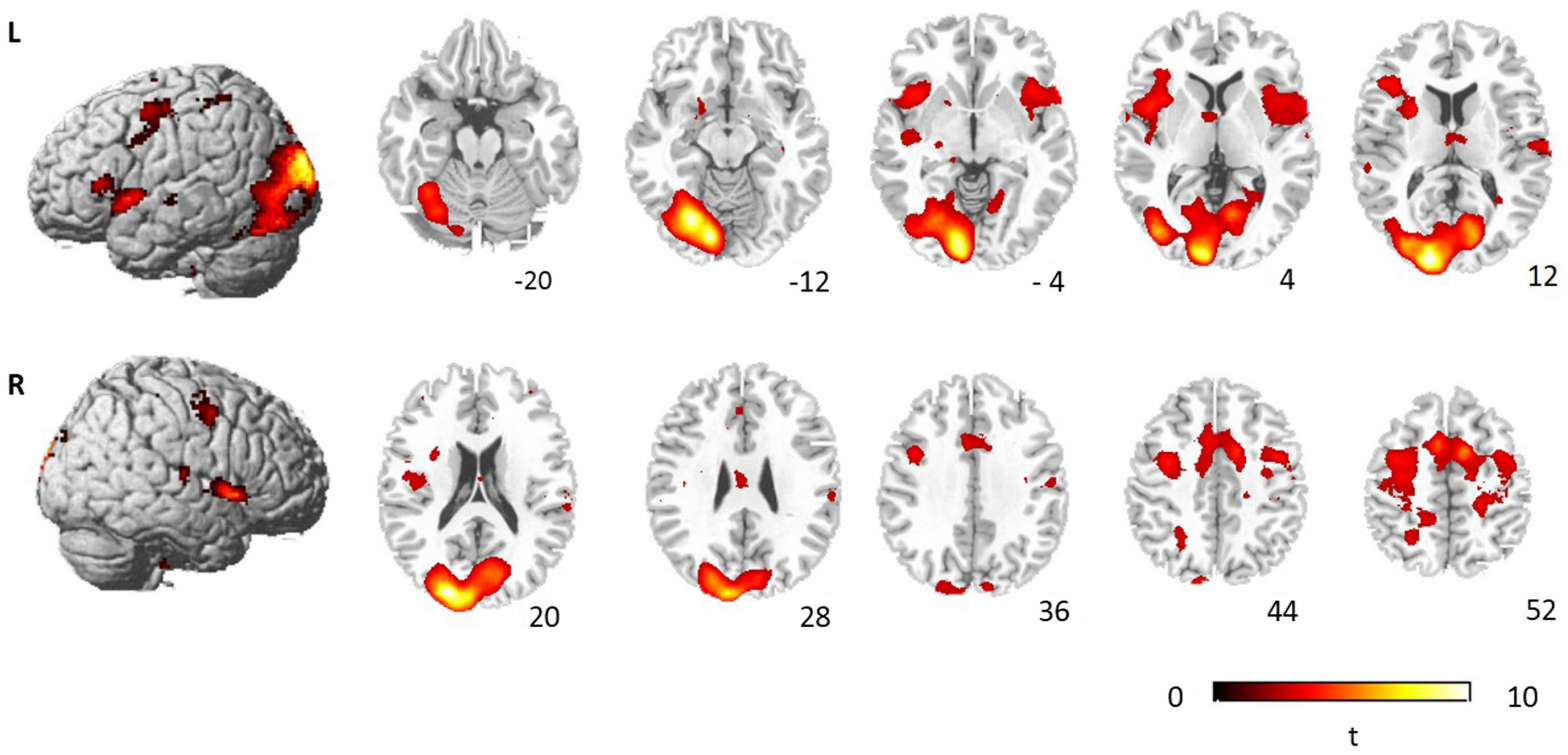
Brain regions showing significant activation during incongruent judgment. The significant threshold is *p* < 0.05 FDR correction.

To examine the neural systems mediating the interference processing, we contrasted brain activation during incongruent condition and neutral condition (Figure 3; Table 2). Significantly activated brain regions comprised bilateral precentral gyrus (BA4/6/7), right medial frontal gyrus (BA6), right MFG (BA6), right paracentral gyrus (BA3), bilateral cingulate gyrus (BA24/31/32), bilateral insula, bilateral STG (BA22), left precuneus (BA7), left lingual gyrus (BA18), left cuneus (BA17), left parahippocampal gyrus (BA28), left cerebellum, left lentiform nucleus and right thalamus. We also compared brain activation between incongruent conditions and congruent conditions, but no significant activation was found after correction for multiple comparisons.

**Table 2:** Coordinates of activation peaks during incongruent decision minus neutral decision.

Brain region	BA	Coordinates (MNI)			peak *Z*
		*x*	*y*	*z*	
Frontal lobe					
L precentral gyrus	4	−32	−12	48	3.89
	6	−38	−4	54	4.04
		−12	−88	44	3.38
L cingulate gyrus	32	−8	10	50	4.46
		−26	−6	46	3.92
L insula	13	−38	12	0	3.98
	13	−44	6	4	3.95
	13	−34	−8	24	3.34
	13	−44	−14	22	3.14
R medial frontal gyrus	6	12	−4	58	3.99
R middle frontal gyrus	6	42	−6	52	3.38
R precentral gyrus	6	40	0	46	3.71
	4	36	−4	60	3.19
R cingulate gyrus	31	22	−34	50	3.73
	24	12	4	50	4.70
R insula	13	46	12	0	3.80
	13	40	18	−2	3.69
	13	50	−14	14	3.09
Temporal lobe					
L superior temporal gyrus	22	−48	−16	−2	3.13
R superior temporal gyrus	22	58	16	−4	3.39
	42	62	−16	10	3.25
Parietal lobe					
L precuneus	7	−22	−38	56	3.76
	7	−22	−48	62	3.56
	7	−16	−44	48	3.43
	7	−26	−60	42	3.04
R postcentral gyrus	3	20	−32	62	3.27
	3	30	−30	60	3.12
R precuneus	7	14	−46	60	3.10
Occipital lobe					
L lingual gyrus	18	−12	−84	−8	7.27
L cuneus	17	−18	−92	14	6.65
Subcortical regions					
L parahippocampal gyrus	28	−26	−20	−10	3.15
L cerebellum		−26	−74	−12	6.39
L lentiform nucleus		−26	8	12	4.02
		−20	10	−10	2.95
R thalamus		6	−12	14	2.88

L, left hemisphere; R, right hemisphere.

**Figure 4:**
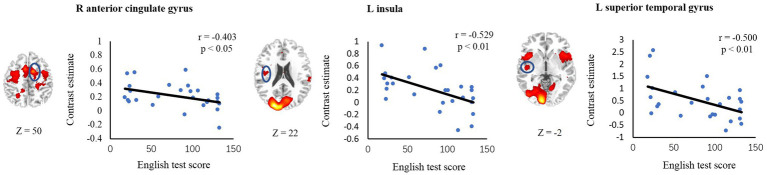
Correlations between brain activity and L2 reading scores. Axial sections and scatter plots for ROIs (in blue circles) showing significant negative correlation are displayed.

To examine the relationship between individual variability in activation levels of inhibitory-related regions and L2 vocabulary proficiency, we conducted correlation analyses for the ROIs based on the intensity of activation during interference processing. Six ROIs, including right MFG, right ACC, left insula, right insula, left STG and left PPC were defined in all-subject incongruent > neutral activation map. This analysis revealed that English test score was negatively correlated with activation levels in the right ACC (*r* = −0.403, *p* < 0.05), left insula (*r* = −0.529, *p* < 0.01) and left STG (*r* = −0.500, *p* < 0.01). However, no significant correlation was discovered in left PPC (*r* = −0.067, *p* = 0.741), right MFG (*r* = −0.050, *p* = 0.804), or right insula (*r* = −0.294, *p* = 0.137; Figure 4).

To further examine whether the correlations remained significant after controlling for the behavioral performance in the conflict task, we conducted correlation analyses between ROI activations and L2 vocabulary proficiencies after partialing out effect of accuracy rates or RTs of the incongruent condition. The partial correlation coefficient controlling for the effects of both Accuracy and RT was −0.409 (*p* < 0.05) for right ACC, −0.539 (*p* < 0.01) for left insula and − 0.241 (*p* = 0.245) for left STG. These results suggest that the activity level in the ACC and insula, which have been consistently found to mediate inhibitory control, were reliably correlated with reading ability of L2.

## Discussion

This study provides an insight into neural plastic changes associated with inhibitory control in bilinguals as a function of L2 proficiency. The Simon effect is widely described as a combination of facilitation for congruent trials and inhibition for incongruent trials. In the neutral trials, responses are not affected by either compatible, or incompatible with respect to the relevant stimulus feature (Masaki et al., 2007; Ferraro et al., 2011). Hence, the difference in brain activation between incongruent condition and neutral condition is an index of inhibition. We found that bilinguals with higher L2 vocabulary proficiency performed better in the Simon task, and more importantly, higher L2 vocabulary proficiency was associated with less involvement of brain regions that support more general cognitive control, including the right ACC, left insula and left STG. Our findings suggested that more frequent use of second language leads to greater neurocognitive advantage, which may be result from a more automatic processing in conflict processing. The finding is consistent with the notion that bilinguals adapted better to conflicting situation and use the brain regions for conflict processing more efficiently than monolinguals (Abutalebi et al., 2012).

We found significant correlations between L2 proficiency and activity level the right ACC, left insula and left STG during inhibitory processing. These brain regions are well-known to serve important parts in cognitive control. The ACC is implicated in monitoring or detecting the occurrence of conflict between task-relevant and task-irrelevant information and subsequently conveys the information to other region to trigger control adjustments (Barch, 2001; Botvinick et al., 2001; Carter and van Veen, 2007). The anterior insula may play a role in monitoring and modulated by error awareness in the conflicting task (Sridharan et al., 2008). In bilinguals, the STG has been recruited for effective interference suppression and decision to respond lies in choosing one of the two (or multiple) conflicting information based on contextual cues (Bialystok et al., 2005; Luk et al., 2010; Mohades et al., 2014).

In multilingual speakers, the aforementioned brain areas, as well as regions such as caudate and dorsolateral prefrontal cortex, are also found to be important for controlling language use (Smith et al., 1996; Reuter-Lorenz et al., 2000; Crinion et al., 2006; Abutalebi et al., 2012). For example, the ACC has been consistently found to activate during a language switching task, in which bilinguals are asked to switch from one language to the other (Wang et al., 2007; Abutalebi et al., 2012). These studies have generally suggested that brain areas for language control in bilinguals overlapped substantially with the brain substrates for general cognitive control.

We found higher L2 vocabulary proficiency in the bilinguals was associated with less involvement of the right ACC, left insula and left STG during the interference processing. A possible interpretation for the negative correlations is that the higher proficient L2 speakers may resolve the conflict more automatically, whereas low proficient L2 speakers recruit cognitive control network to a greater extent to inhibit interference items during the task. Previous studies have demonstrated that automatic inhibition can develop over practice (Schneider and Shiffrin, 1977; Verbruggen and Logan, 2008). Constantly resolving language conflicts in higher proficient bilinguals may lead to more automatic inhibition of a non-target language. Such change may also affect the way in which the brain deals with general cognitive control. Future studies using longitudinal design are needed to elucidate the exact mechanisms by which L2 acquisition affects cognitive control in the bilingual brain. Despite that, some studies have implied that while bilinguals have reduced task-based activity, the functional connectivity between areas was generally higher (Costumero et al., 2015; Kousaie et al., 2017; Gullifer et al., 2018). According to these studies, we wonder whether a change in the pattern of functional connectivity exist and could explain our results. We conducted a connectivity analysis on the defined ROIs (i.e., right ACC, left insula and left STG). The connectivity between the ROIs showed significant correlation between them [i.e., *r* (ACC, insula) = 0.50, *p* < 0.01; *r* (ACC, STG) = 0.55, *p* < 0.01; *r* (insula, STG) = 0.76, *p* < 0.01]. However, we only detected effects of L2 proficiency and inhibitory reaction (i.e., RT and Accuracy) on the connectivity of defined ROIs. Future research is needed to examine whether connections between other regions were modulated by L2 proficiency.

The differences of mean RTs between three conditions reflected the conflict effect and facilitate effect. Interestingly, the difference among three conditions did not achieve significant. These results are quite consistent with the previous findings obtained with the Simon tasks and integrated Simon Stroop tasks in adults (Liu et al., 2004; Wang et al., 2013). These tasks consistently instructed participants to respond while ignoring the location of the stimulus. There might be a relatively fixed reaction time although it was influenced by confliction or facilitation. The age of participants might be another possible contributor. Adults might have better control ability under the conflict effect or facilitate effect, which caused lesser impact on stimuli-reaction. In addition, our participants accepted higher education and had steady job, whose experiences might assist in staying relatively steady stimuli-reaction. Meanwhile, present result showed that RTs were not influenced by English test scores, ages of acquisition English or ages. It suggested that these factors might cause similar RTs when the participants complete the judgment task.

Results from the present study have generated evidence indicating that the L2vocabulary proficiency contributes to inhibitory control. The participants, who had the similar background (i.e., the linguistic background, socioeconomic status, handness, and education experience), were over 30 years older. And we have confirmed that the correlation between L2 vocabulary proficiency and inhibitory control ability were not disturbed by age in this group. In the following study, longitudinal design is needed to elucidate the relationship in different age groups.

At present other factors (i.e., listening, speaking and writing) associated with the second language ability were not examined. In addition, our test just detected vocabulary size and word frequency effects. The number of associations and within-group consistency of participants’ associative domain are also known as better predictors of language proficiency. Future research might provide a deeper understanding of the relationship between L2 proficiency and inhibitory control ability by including more factors related to second language ability.

## Data Availability Statement

The datasets presented in this article are not readily available because the data that support the findings of this study are available from the corresponding author upon reasonable request. Requests to access the datasets should be directed to spe_jiafl@ujn.edu.cn.

## Ethics Statement

The studies involving human participants were reviewed and approved by ethical committee of the Beijing MRI Center for Brain Research, Chinese Academy of Sciences. The patients/participants provided their written informed consent to participate in this study.

## Author Contributions

FJ conceived and designed the experiment. FJ wrote the paper and performed the data analyses.

## Funding

This work was supported by National Natural Science Foundation of China (Nos. 32100856, 32171054, and 31800913), Guangdong Pearl River Talents Plan Innovative and Entrepreneurial Team grant (2016ZT06S220), Project of Humanities and Social Sciences Financed by Ministry of Education (21YJC880028), and Doctoral Fund of the University of Jinan (No. 160100446).

## Conflict of Interest

The authors declare that the research was conducted in the absence of any commercial or financial relationships that could be construed as a potential conflict of interest.

## Publisher’s Note

All claims expressed in this article are solely those of the authors and do not necessarily represent those of their affiliated organizations, or those of the publisher, the editors and the reviewers. Any product that may be evaluated in this article, or claim that may be made by its manufacturer, is not guaranteed or endorsed by the publisher.
